# Nrf2 as a Molecular Guardian of Redox Balance and Barrier Integrity in IBD

**DOI:** 10.3390/antiox14121407

**Published:** 2025-11-26

**Authors:** Bojan Stojanovic, Ivana Milivojcevic Bevc, Milica Dimitrijevic Stojanovic, Bojana S. Stojanovic, Marina Jovanovic, Snezana Lazarevic, Bojan Milosevic, Ivan Radosavljevic, Danijela Tasic-Uros, Nenad Markovic, Đorđe Todorovic, Irfan Corovic, Filip Milutinovic, Damnjan Pantic, Mladen Maksic, Snezana Lukic

**Affiliations:** 1 Department of Surgery, Faculty of Medical Sciences, University of Kragujevac, 34000 Kragujevac, Serbia; bojan.stojanovic01@gmail.com (B.S.);; 2 Center for Molecular Medicine and Stem Cell Research, Faculty of Medical Sciences, University of Kragujevac, 34000 Kragujevac, Serbia; 3 City Medical Emergency Department, 11000 Belgrade, Serbia; 4 Department of Pathology, Faculty of Medical Sciences, University of Kragujevac, 34000 Kragujevac, Serbia; 5 Department of Pathophysiology, Faculty of Medical Sciences, University of Kragujevac, 34000 Kragujevac, Serbia; 6 Department of Internal Medicine, Faculty of Medical Sciences, University of Kragujevac, 34000 Kragujevac, Serbia; 7 Department of Neurology, Faculty of Medical Sciences, University of Kragujevac, 34000 Kragujevac, Serbia; 8 Department of Radiology, Faculty of Medical Sciences, University of Kragujevac, 34000 Kragujevac, Serbia

**Keywords:** Nrf2, oxidative stress, inflammatory bowel disease, intestinal barrier, redox signaling

## Abstract

Inflammatory bowel disease (IBD), encompassing Crohn’s disease and ulcerative colitis, represents a chronic inflammatory condition of the gastrointestinal tract characterized by immune dysregulation, oxidative stress, and impaired epithelial barrier function. Among the complex molecular networks involved, the transcription factor nuclear factor erythroid 2–related factor 2 (Nrf2) has emerged as a central regulator of redox balance, anti-inflammatory signaling, and mucosal homeostasis. This review provides a comprehensive overview of the pathogenic role of oxidative stress in IBD, detailing the sources and consequences of reactive oxygen species (ROS) accumulation, and the mechanisms by which Nrf2 activation counters these effects. We discuss the dual regulation of Nrf2 through Keap1-dependent and Keap1-independent pathways, its influence on epithelial integrity, immune cell polarization, microbiota composition, and its paradoxical role in IBD-associated tumorigenesis and fibrosis. Furthermore, we highlight emerging therapeutic strategies aimed at modulating Nrf2 activity via pharmacologic agents, dietary phytochemicals, and probiotics. Taken together, these insights position Nrf2 as a pivotal therapeutic target in IBD, offering new avenues for restoring epithelial resilience, mitigating chronic inflammation, and improving clinical outcomes.

## 1. Introduction

Inflammatory bowel disease represents a chronic relapsing inflammatory disorder of the gastrointestinal tract driven by complex interactions among genetics, immunity, microbiota, and environmental influences. Despite significant advances in understanding immune and microbial mechanisms, the contribution of oxidative stress and its regulatory network, particularly the Nrf2/Keap1 signaling axis, remains incompletely defined. The existing literature highlights oxidative stress as a key amplifier of mucosal injury and immune dysregulation, yet the precise molecular links between redox imbalance, epithelial barrier failure, immune modulation, and intestinal fibrosis or tumorigenesis are insufficiently integrated. This review aims to synthesize current experimental and clinical evidence on the Nrf2-centered redox network in IBD, outlining its crosstalk with inflammatory and metabolic pathways, its physiological and pathological roles in intestinal homeostasis, and its translational relevance. To achieve this, comprehensive literature analysis was performed using recent peer-reviewed publications, with emphasis on mechanistic, preclinical, and therapeutic data that collectively illustrate the evolving understanding of Nrf2 as a promising target in IBD management.

## 2. IBD at a Glance: Phenotypes, Diagnosis, and Global Burden

Inflammatory bowel disease comprises two related but distinct entities: Crohn’s disease (CD) and ulcerative colitis (UC), that present with chronic, relapsing inflammation of the gastrointestinal tract [1]. The hallmark that separates them is the depth and distribution of tissue injury. CD produces transmural inflammation that can affect any segment from mouth to anus, with a predilection for the terminal ileum [2]. UC is limited to the colon and primarily involves the mucosa in a continuous pattern starting from the rectum [3]. These anatomic and histologic differences underlie divergent clinical courses and complications [4].

Diagnosis rests on the integration of clinical assessment with endoscopy, imaging, and histopathology [4]. Endoscopy and biopsies define lesion distribution and depth, while cross-sectional imaging captures transmural damage and skip lesions typical of CD [5,6,7]. Systemic inflammation can extend beyond the gut and manifest as extraintestinal features, emphasizing that IBD is a multisystem disease [8]. In practice, the mechanistic link is straightforward: sustained mucosal injury in UC drives bloody diarrhea and urgency, whereas transmural penetration in CD fosters strictures, fistulas, and abscesses [9].

IBD can occur at any age, but both disorders most often debut in late adolescence or early adulthood [10]. UC is frequently diagnosed several years later than CD on average [11]. This age pattern has clinical implications: early-life onset coincides with critical periods for growth, education, and family planning, magnifying disease burden even when symptoms wax and wane [12].

Epidemiological studies reveal that the rates are high and continue to rise across the globe [13]. While Western nations still account for the highest overall prevalence, affecting roughly 0.3% of the population, the most rapid growth is now being observed in newly industrialized regions such as East Asia [14]. The burden remains significant in countries with high and medium–high socio-demographic indices and is steadily increasing in those with low and medium indices [15]. This trend reflects broader global changes in environmental conditions, lifestyle patterns, and access to healthcare [16,17].

Outcomes mirror underlying pathology. CD carries higher all-cause mortality than the general population and often necessitates surgery; over a lifetime, most patients require at least one intestinal resection, and a subset will need a permanent stoma [18,19]. UC, while confined to the colon, still leads 10–30% of patients to colectomy [20]. Chronic, uncontrolled inflammation in both conditions increases the risk of intestinal cancer, consistent with the inflammation–dysplasia–carcinoma sequence [21,22]. These trajectories underscore a central principle: the depth and extent of inflammation shape complications, surgical needs, and long-term malignancy risk, guiding surveillance and therapeutic strategy [23,24].

### Etiology and Treatment Landscape of IBD: Interplay of Genetics, Immunity, Microbiota, and Redox Control

Inflammatory bowel disease arises from a multifactorial process in which genetic susceptibility interacts with immune dysregulation, environmental exposures, and intestinal dysbiosis; the precise initiating cause remains undefined [17,25]. Genome-wide studies implicate over a hundred risk loci that converge on pathways governing innate and adaptive immunity, epithelial barrier function, microbial sensing, and cellular stress responses, including oxidative and electrophilic stress [17,26]. Variants affecting redox control (e.g., regulators of antioxidant defenses and electrophile response) can lower the threshold for tissue injury, intensify cytokine signaling, and weaken mucosal repair, thereby amplifying inflammation [27,28].

The gut microbiota serves as both trigger and amplifier of disease activity [29]. Microbial antigens and metabolites influence the host immune system by engaging pattern recognition receptors and metabolic checkpoints [30]. At the same time, environmental factors such as diet, smoking, medications, and early-life exposures like breastfeeding alter the composition and activity of the gut microbiota [31,32]. These influences can modify gene expression via epigenetic mechanisms (DNA methylation, histone marks, and microRNAs), linking external cues to persistent changes in epithelial and immune cell behavior [33,34]. The result is a self-reinforcing loop in which barrier defects permit microbial translocation, innate and adaptive responses escalate, and redox imbalance (excess reactive oxygen species with inadequate antioxidant compensation) sustains tissue damage [27,35]. 

**Figure 1:**
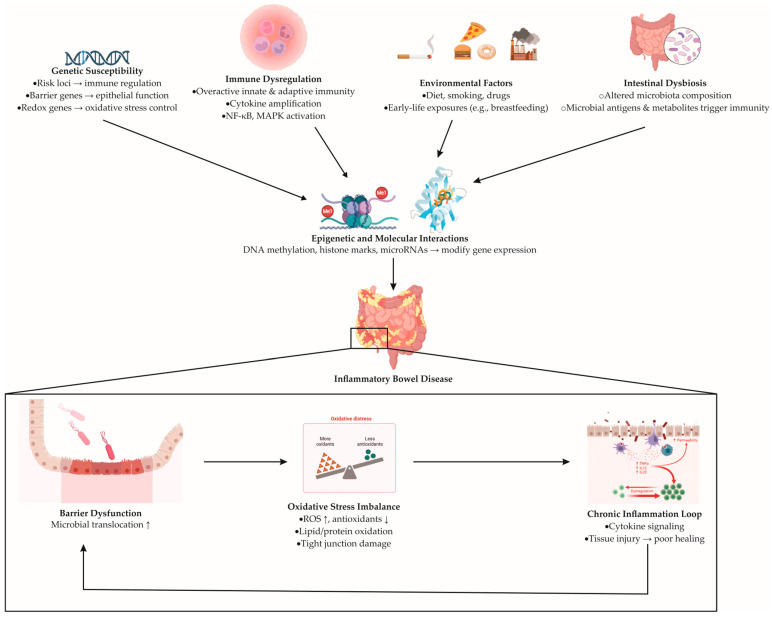
Proposed mechanisms underlying the pathogenesis of inflammatory bowel disease. Genetic susceptibility (risk variants affecting immune regulation, epithelial barrier genes, and redox-related genes), immune dysregulation (overactivation of innate and adaptive immunity), environmental factors (diet, smoking, medications, early-life exposures), and intestinal dysbiosis (altered gut microbiota composition and microbial metabolites) converge to induce epigenetic and molecular alterations (DNA methylation, histone modifications, and microRNA regulation). These processes drive persistent immune activation and epithelial dysfunction, ultimately leading to IBD development. The downstream cycle includes barrier disruption, oxidative stress imbalance, and chronic inflammation. Barrier injury promotes microbial translocation; oxidative stress—characterized by increased ROS (reactive oxygen species) and reduced antioxidants—amplifies cytokine signaling via NF-κB (nuclear factor kappa-light-chain-enhancer of activated B cells) and MAPK (mitogen-activated protein kinase) pathways, while inadequate antioxidant defenses impair tissue repair.

Within this network, oxidative stress is not merely a by-product but a driver of pathophysiology [35]. Reactive oxygen species produced by activated immune cells and stressed epithelial cells oxidize lipids and proteins, weaken tight junctions, and amplify pro-inflammatory signaling pathways, including nuclear factor kappa B (NF-κB) and mitogen-activated protein kinase (MAPK) [36,37]. In contrast, protective cellular mechanisms, especially those regulated by electrophile and antioxidant sensors, support the detoxification of harmful compounds, maintain the balance of glutathione, and help bring inflammation to an end [38,39]. When genetic variation or environmental pressure blunts these defenses, inflammation persists and healing is delayed. The multifactorial pathogenesis of inflammatory bowel disease, illustrating the interplay between genetic, immune, microbial, and environmental determinants, is schematically summarized in Figure 1.

Current therapy targets immune activation and mucosal injury but remains imperfect [40]. Standard regimens include aminosalicylates, corticosteroids, immunosuppressants, and biologics aimed at key cytokines or trafficking pathways [41]. Although effective for induction and maintenance in many patients, these agents are limited by primary non-response, loss of response, adverse effects, and treatment-limiting toxicity [42]. These constraints underscore the need for strategies that also restore barrier function and rebalance redox and epithelial stress pathways—complementing immunomodulation to achieve durable remission.

## 3. Oxidative Stress: Sources, Molecular Targets, and Antioxidant Control

Oxidative stress is a disruption of cellular redox balance that occurs when the generation of reactive oxygen species (ROS) exceeds the capacity of antioxidant defenses to neutralize them [43]. ROS comprise radical and non-radical oxygen-containing species produced continuously in aerobic cells, with mitochondria as a major source during oxidative phosphorylation [43]. At physiological levels, ROS function as second messengers that tune pathways governing proliferation, differentiation, apoptosis, host defense, and inflammation [44]. When ROS accumulate, their high reactivity drives indiscriminate chemistry with nearby macromolecules, damaging membrane lipids, proteins, and nucleic acids and disturbing membrane fluidity and cell volume regulation [45].

The principal pro-oxidant species include superoxide (O_2_•^−^), hydrogen peroxide (H_2_O_2_), and the hydroxyl radical (•OH) [45]. Other important contributors include singlet oxygen, hypochlorous acid (HOCl), ozone (O_3_), chloramines, and lipid peroxyl and hydroperoxide species [46]. Many of these reactive molecules are produced through enzymatic reactions in immune cells, such as those involving nicotinamide adenine dinucleotide phosphate (NADPH) oxidases and myeloperoxidase, or through metal-catalyzed redox cycling [47]. A moderate level of ROS supports the destruction of microbiota, wound healing, and tissue repair [48,49,50]. However, when ROS production becomes excessive, it overwhelms the body’s detoxification systems, promotes lipid peroxidation and protein carbonylation, causes DNA strand breaks and base alterations, and activates pro-inflammatory signaling pathways that worsen tissue damage [51].

Cells counterbalance these pressures through layered antioxidant networks. Enzymatic defense systems form the main pathway for detoxification [52]. Superoxide dismutases convert superoxide anions into hydrogen peroxide, while catalase and glutathione peroxidases further break down hydrogen peroxide and lipid hydroperoxides into water or their corresponding alcohols [53,54,55]. These reactions rely on catalytic heme groups or glutathione as an electron donor [53,54,55]. Non-enzymatic antioxidants complement these enzymes by buffering electrophiles and free radicals in cellular and extracellular spaces [39]. Key molecules include reduced glutathione (GSH), ascorbate (vitamin C), and α-tocopherol (vitamin E), which participate in tightly coupled recycling cycles that preserve redox homeostasis [39,56].

In the intestine, persistent exposure to luminal microbiota and diet-derived oxidants imposes recurrent redox stress [35]. When ROS generation in inflamed mucosa outpaces local antioxidant capacity, oxidative damage extends beyond the epithelium into deeper layers of the bowel wall [57,58]. The resulting loss of barrier integrity, accumulation of oxidized lipids and proteins, and activation of redox-sensitive inflammatory pathways collectively shift the tissue from controlled signaling to chronic injury, setting the stage for sustained immune activation characteristic of inflammatory bowel disease [27].

### 3.1. Physiological Roles of Free Radicals and Reactive Species

Although often associated with tissue damage, free radicals are indispensable for maintaining normal physiological functions [51]. When generated at controlled levels, ROS and RNS act as essential mediators in cellular and systemic homeostasis [38,59]. In the immune system, for instance, phagocytes such as neutrophils and macrophages deliberately produce superoxide and other radicals via the NADPH oxidase complex [60]. These reactive species are stored and released during phagocytosis to destroy invading microorganisms [60]. The clinical importance of this mechanism is evident in chronic granulomatous disease, where defective NADPH oxidase activity prevents superoxide formation, resulting in recurrent and persistent infections [61].

Beyond their antimicrobial function, free radicals contribute to intracellular signaling networks. Nonphagocytic isoforms of NADPH oxidase generate low levels of ROS in fibroblasts, endothelial cells, vascular smooth muscle cells, cardiac myocytes, and thyroid tissue, where they serve as second messengers that fine-tune gene expression, proliferation, and adaptive responses [62]. Among these signaling molecules, NO is the most recognized. Produced by nitric oxide synthases, NO regulates vascular tone and platelet aggregation, supports synaptic transmission in the nervous system, and mediates cytotoxic defense against pathogens and tumor cells [63,64]. Furthermore, moderate ROS generation can activate mitogenic pathways, promoting cell growth and tissue repair [65].

Thus, while excessive or chronic radical exposure drives oxidative stress and cellular injury, their physiological presence at low concentrations is vital for immune competence, vascular homeostasis, and signal transduction. The balance between beneficial signaling and pathological oxidation underscores the importance of redox regulation in health and disease.

### 3.2. Oxidative Stress as a Central Pathogenic Driver in IBD: Molecular Mechanisms and Clinical Relevance

Redox homeostasis is one of the core biological systems disturbed in inflammatory bowel disease [27]. Under physiological conditions, epithelial and immune cells continually balance oxidant production with enzymatic and non-enzymatic antioxidant defenses [66]. In IBD, this equilibrium collapses [27]. The chronically inflamed mucosa is exposed to oxidants originating from diet, microbiota, and infiltrating neutrophils and macrophages, which generate abundant ROS and reactive nitrogen species [27,67,68]. When antioxidant buffering becomes insufficient, the entire redox network tilts toward oxidative and nitrosative stress, creating an environment that favors tissue injury rather than healing [37,69].

A disrupted redox balance undermines the structural and immunological integrity of the intestinal barrier. Excess ROS not only damage lipids, proteins, and DNA but also reshape epithelial signaling programs [27]. Lipid peroxidation products such as malondialdehyde and 4-hydroxynonenal disturb tight junction organization, increase mucosal permeability, and facilitate bacterial translocation [70,71]. As epithelial defenses weaken, luminal antigens gain access to immune compartments, amplifying Toll-like receptor signaling and promoting sustained recruitment of neutrophils and monocytes [27]. This establishes a self-reinforcing inflammatory loop: barrier leakage increases antigen load, immune activation escalates, and ROS generation rises even further.

Protein and DNA oxidation add another layer of dysfunction [72,73]. Oxidative modifications impair protein structure, enzyme activity, and cellular redox sensors, while DNA lesions interfere with cell-cycle regulation and increase mutational burden—mechanisms that partly explain the heightened risk of dysplasia and colorectal cancer in long-standing IBD [27].

At the signaling level, oxidative stress acts as both a trigger and amplifier of inflammation [27]. Nuclear factor κB and MAPKs act as central regulators that translate oxidative signals into gene expression programs, driving the production of cytokines, chemokines, adhesion molecules, and enzymes such as inducible nitric oxide synthase (iNOS) and myeloperoxidase [47,74]. This gene expression pattern sustains recruitment of neutrophils and monocytes and reinforces local production of ROS/RNS, cementing chronic inflammation [74]. At the same time, the cytokine environment shifts toward tumor necrosis factor alpha (TNF-α), interleukin 1 beta (IL-1β), and interleukin 8 (IL-8), which further enhance the recruitment of immune cells and intensify epithelial stress [75].

In parallel, the Nrf2 pathway—responsible for upregulating antioxidant and cytoprotective genes—is often insufficiently activated or overwhelmed during active disease, further widening the gap between oxidant load and defensive capacity [76]. The interplay between NF-κB–driven inflammation and inadequate Nrf2-mediated counter-regulation forms a molecular core of redox imbalance in IBD [27].

**Figure 2:**
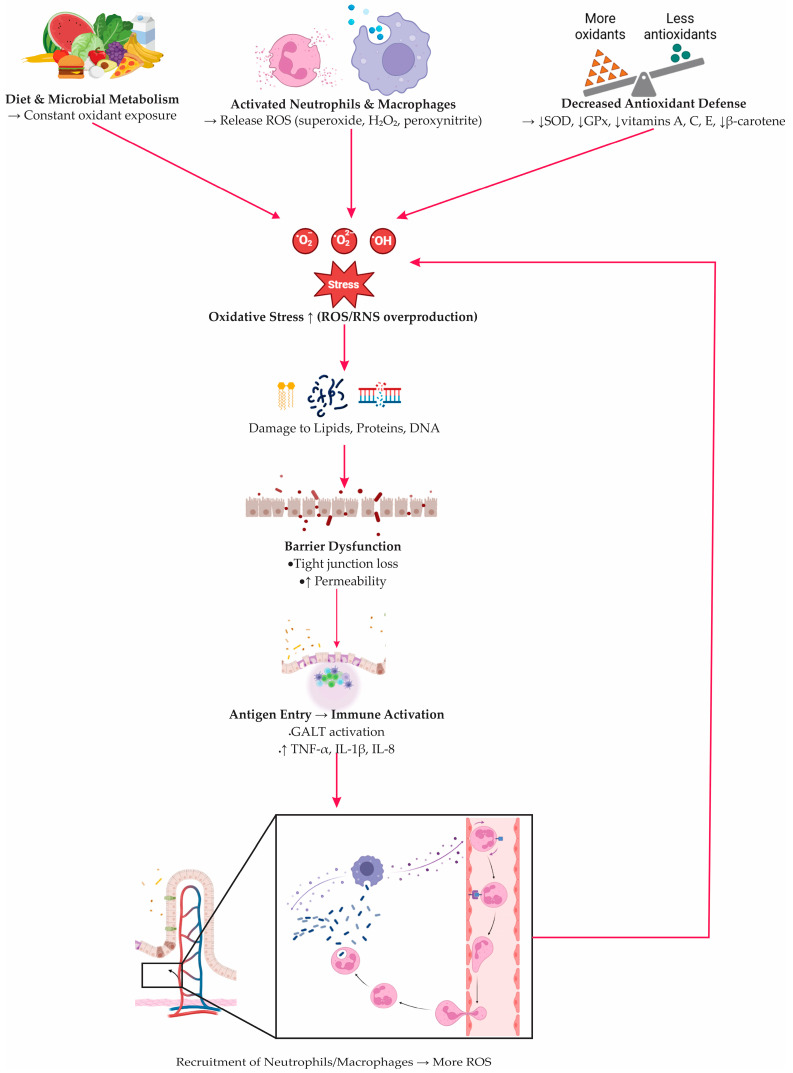
Oxidative stress–driven epithelial injury and immune activation in inflammatory bowel disease. Dietary and microbial oxidants, together with ROS/RNS released by activated neutrophils and macrophages (ROS: reactive oxygen species; RNS: reactive nitrogen species), overwhelm epithelial redox balance due to reduced antioxidant defenses (SOD: superoxide dismutase; GPx: glutathione peroxidase; vitamins A, C, E, β-carotene). Excess oxidants damage lipids, proteins, and DNA, causing tight-junction loss and increased permeability. Translocated antigens activate GALT (gut-associated lymphoid tissue) and stimulate cytokine production (TNF-α: tumor necrosis factor-alpha; IL-1β: interleukin-1β; IL-8: interleukin-8), promoting further immune-cell recruitment and reinforcing the oxidative–inflammatory loop.

Evidence from patients supports this mechanism. The activities of superoxide dismutase and glutathione peroxidase are often diminished in both the intestinal mucosa and the blood [77,78,79]. At the same time, levels of non-enzymatic antioxidants such as vitamins A, C, and E, as well as beta-carotene, are reduced, reflecting an overall imbalance in the body’s redox status [66,79]. Oxidative injury markers extend beyond the bowel, with elevated H_2_O_2_ detected in circulating monocytes and lymphocytes during active disease [79]. Clinical indicators reflect these biological changes. Composite measures of oxidative stress show a positive correlation with C-reactive protein (CRP) and fibrinogen, while reduced levels of plasma free thiols, which are important substrates for ROS, are associated with more severe inflammation and worse clinical outcomes [78,79]. The cascade of oxidative stress–driven mucosal injury, illustrating the interplay between oxidant overproduction, barrier failure, and immune activation, is summarized in Figure 2.

## 4. Nrf2–Keap1 Pathway: Central Regulator of Antioxidant and Anti-Inflammatory Defense

**Figure 3:**
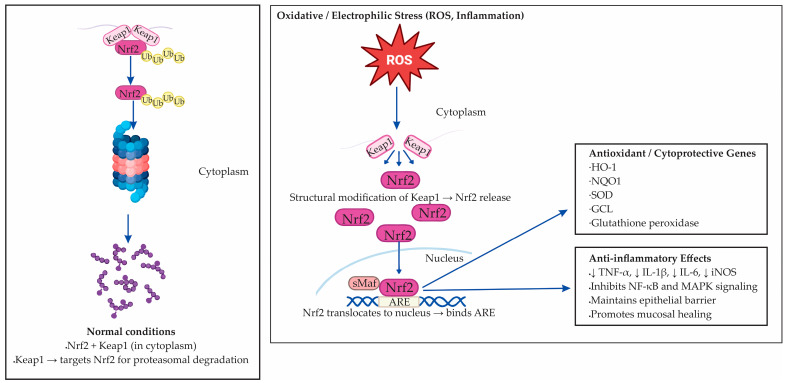
The Nrf2–Keap1 signaling pathway in oxidative and electrophilic stress. Under normal conditions, nuclear factor erythroid 2–related factor 2 (Nrf2) remains bound to Kelch-like ECH-associated protein 1 (Keap1) in the cytoplasm, where Keap1 targets Nrf2 for ubiquitination and proteasomal degradation. Upon exposure to oxidative or electrophilic stress, structural modification of Keap1 cysteine residues causes Nrf2 release and nuclear translocation. In the nucleus, Nrf2 forms heterodimers with small Maf proteins and binds to antioxidant response elements (AREs), initiating transcription of cytoprotective and antioxidant genes, including heme oxygenase-1 (HO-1), NAD(P)H:quinone oxidoreductase 1 (NQO1), superoxide dismutase (SOD), glutamate–cysteine ligase (GCL), and glutathione peroxidase. Activation of Nrf2 also suppresses pro-inflammatory mediators (TNF-α, IL-1β, IL-6, iNOS), inhibits NF-κB and MAPK signaling, preserves epithelial barrier integrity, and promotes mucosal healing.

The Nuclear factor erythroid 2–related factor 2 (Nrf2 ) is a master transcription factor responsible for coordinating the cellular antioxidant response to oxidative and electrophilic stress [80]. Under normal conditions, Nrf2 is bound in the cytoplasm to Kelch-like ECH-associated protein 1 (Keap1), which targets it for degradation by the proteasome [81]. When cells are exposed to stress signals such as ROS, electrophilic compounds, or metabolites generated during inflammation, structural changes occur in Keap1 that release Nrf2 [82]. The liberated Nrf2 then moves into the nucleus to initiate protective gene expression. There, Nrf2 binds to antioxidant response elements (AREs) in the promoters of target genes, initiating the transcription of cytoprotective molecules [82,83]. The dual role of the Nrf2–Keap1 signaling axis under basal and stress conditions, and its downstream antioxidant and anti-inflammatory targets, is illustrated in Figure 3.

Nrf2 activation leads to upregulation of a broad range of enzymes involved in redox balance, detoxification, and cellular repair [82,84]. These include heme oxygenase-1 (HO-1), NAD(P)H:quinone oxidoreductase 1 (NQO1), superoxide dismutase (SOD), glutamate–cysteine ligase (GCL), and glutathione peroxidase. Together, they re-establish glutathione homeostasis, neutralize ROS, and prevent further oxidative damage [82,84]. In parallel, Nrf2 suppresses the expression of pro-inflammatory cytokines such as TNF-α, IL-1β, IL-6, and iNOS, thereby exerting anti-inflammatory effects [85].

The relevance of Nrf2 extends beyond oxidative detoxification. Experimental evidence shows that activation of Nrf2 can suppress NF-κB signaling, maintain the integrity of the epithelial barrier, and support mucosal regeneration [86,87]. These functions are particularly relevant to the pathogenesis and treatment of IBD [87]. In murine models and human samples, enhanced Nrf2 activity correlates with reduced inflammatory infiltration and improved histological scores [88,89,90]. Conversely, impaired Nrf2 signaling is associated with sustained oxidative damage, increased permeability of the intestinal barrier, and exaggerated immune responses [87,90].

Beyond the gut, the Nrf2 pathway has shown therapeutic promise in other inflammation-driven conditions, including neurodegeneration, aging, and carcinogenesis [91,92,93]. Its dual capacity to neutralize ROS and modulate immune signaling makes Nrf2 a strategic target in diseases characterized by chronic inflammation and redox imbalance [82]. In IBD specifically, pharmacological or dietary activation of Nrf2 may complement conventional immunosuppressive therapies by reinforcing the mucosal antioxidant shield and breaking the vicious cycle of oxidative stress and immune activation [94,95].

### 4.1. Structural Complexity and Activation Mechanism of Nrf2 in Redox Regulation

**Figure 4:**
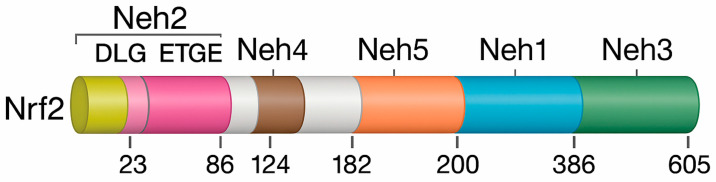
Structural organization of human Nrf2 and its conserved Neh domains. Nrf2 contains seven Nrf2–ECH homology (Neh) domains with distinct regulatory functions. Neh2 at the N-terminus includes the DLG and ETGE motifs that mediate Keap1 binding. Neh4 and Neh5 function as transactivation modules, while Neh1 contains the bZIP (basic leucine zipper) region required for small Maf heterodimerization and ARE binding. Neh3 at the C-terminus contributes to transcriptional activation. Neh6 regulates Keap1-independent degradation via β-TrCP, and Neh7 represses Nrf2 activity through interaction with RXRα (retinoid X receptor-alpha).

Nuclear factor erythroid 2–related factor 2, encoded by the *NFE2L2* gene, is a central transcriptional regulator of antioxidant defense and redox balance [96]. Originally cloned from the human leukemia K562 cell line, Nrf2 belongs to the Cap’n’Collar (CNC) subfamily of basic leucine zipper (bZIP) transcription factors, which also includes Nrf1, Nrf3, and the NF-E2 p45 subunit [97,98,99]. The human Nrf2 protein consists of 605 amino acids and contains seven highly conserved functional domains, known as Nrf2 –ECH homology (Neh1–Neh7), each of which plays a specific role in regulating the stability, localization, and transcriptional activity of the protein [100,101]. The modular organization of Nrf2 and its conserved Neh domains, which mediate DNA binding, protein stability, and transcriptional activation, is illustrated in Figure 4.

The Neh1 domain contains the bZIP structure required for dimerization with small musculoaponeurotic fibrosarcoma (Maf) proteins and DNA binding [102]. Neh2, located near the N-terminus, is the principal regulatory site for Keap1-dependent ubiquitination via its DLG and ETGE motifs, thereby controlling basal Nrf2 degradation [96,103]. The C-terminal Neh3 domain supports transcriptional activation through interactions with chromatin-remodeling factors such as CHD6, while Neh4 and Neh5 function as transactivation domains that recruit coactivators essential for antioxidant gene induction [96].

Neh6 domain, enriched in serine residues, mediates Keap1-independent proteasomal degradation via β-TrCP [104]. Conversely, Neh7 binds retinoid X receptor-α (RXRα), which suppresses Nrf2 by blocking its transcriptional activity, revealing cross-talk between nuclear receptors and redox pathways [104,105].

Under basal conditions, Nrf2 is sequestered in the cytoplasm by Keap1, which acts as an adaptor for the Cul3–Rbx1 ubiquitin ligase complex that continuously targets Nrf2 for proteasomal degradation [106]. Oxidative or electrophilic stress modifies critical cysteine residues in Keap1, disrupting Keap1–Nrf2 binding and preventing ubiquitination [82,107]. Stabilized Nrf2 accumulates, enters the nucleus, dimerizes with Maf proteins, and binds ARE sequences to initiate transcription [96].

Nrf2 activation induces a broad cytoprotective program, including detoxification enzymes (GST, NQO1), antioxidant systems (SOD, GPx), and HO-1, which collectively enhance resistance to oxidative injury, inflammation, and apoptosis [76,82]. Through this tightly regulated architecture, Nrf2 acts as a central redox-sensing switch that maintains cellular homeostasis—an especially important function in IBD, where Nrf2-mediated defenses may mitigate mucosal damage and chronic inflammation [76].

### 4.2. Mechanisms of Nrf2 Activation: Keap1-Dependent and Keap1-Independent Pathways

Nrf2–ARE signaling can be activated through both Keap1-dependent and Keap1-independent mechanisms, each converging on the induction of cytoprotective genes [81].Under basal conditions, Keap1 binds Nrf2 via its DLG and ETGE motifs and targets it for Cul3–Rbx1–mediated ubiquitination and proteasomal degradation [108,109]. Oxidative or electrophilic stress modifies key cysteine residues of Keap1—particularly Cys151, Cys273, and Cys288—disrupting its E3 ligase activity and allowing Nrf2 to accumulate, translocate to the nucleus, dimerize with small Maf proteins, and activate ARE-driven transcription [80,82,110,111,112,113].

Nrf2 can also be activated independently of Keap1 through phosphorylation by several kinases [114,115]. Protein kinase C (PKC) promotes Nrf2 release and nuclear import via Ser40 phosphorylation [82,116], while adenosine monophosphate–activated protein kinase (AMPK) enhances Nrf2 nuclear accumulation and antioxidant gene expression through Ser550 modification, contributing to improved stress tolerance [82,116,117,118,119,120,121]. Mitogen-activated protein kinases (MAPKs), including extracellular signal–regulated kinase (ERK), c-Jun N-terminal kinase (JNK), and p38—further modulate Nrf2 stability and transactivation potential, although their exact phosphorylation sites are still being defined [119,120]. Together, these pathways integrate redox, metabolic, and stress signals to fine-tune Nrf2 activation and antioxidant defense.

Together, the Keap1-dependent sensor system and Keap1-independent kinase pathways allow for flexible, multilayered regulation of Nrf2. This coordination enables cells to quickly respond to various stress signals, from oxidative injury to metabolic disturbances, by activating a comprehensive antioxidant defense program. The central components and activation routes of the Nrf2–Keap1 pathway, along with their molecular effects and therapeutic relevance in inflammatory bowel disease, are summarized in Table 1.

## 5. Physiological Role of Nrf2 in Intestinal Development and Barrier Function

The Nrf2–ARE signaling pathway plays an essential role not only in cellular redox defense but also in intestinal morphogenesis, epithelial homeostasis, and barrier maintenance [121,122]. During embryonic development, Nrf2 expression is spatially and temporally regulated. Elevated levels of Nrf2 mRNA were detected in the hindgut during late gestation, particularly between days 14.5 and 18.5 of mouse embryogenesis, in contrast to declining expression in organs such as the lung and heart [121,123,124]. This suggests a developmentally restricted role of Nrf2 in intestinal formation. Indeed, Nrf2 transcriptional activity is necessary for the establishment of normal epithelial architecture, including villus formation, crypt development, and lineage specification of absorptive and secretory cells [121,125].

At the molecular level, Nrf2 orchestrates intestinal epithelial differentiation by modulating two key developmental pathways: Notch and Wnt [76]. The Notch1 promoter contains a functional ARE, enabling direct transcriptional regulation by Nrf2 [126]. Loss of Nrf2 impairs Notch1 signaling, which disrupts enterocyte maturation [121]. Similarly, the Notch effector Math1 appears to be indirectly repressed by Nrf2, suggesting a balancing role between proliferation and differentiation [76,127]. On the other hand, Wnt signaling interacts bidirectionally with Nrf2, as β-catenin can activate Nrf2, while Nrf2 negatively regulates β-catenin, creating a feedback loop that fine-tunes epithelial cell fate decisions, especially the differentiation into Paneth cells critical for barrier integrity [94,128,129].

In adult tissue, Nrf2 maintains intestinal barrier function by preserving tight junctions (TJs) and limiting epithelial permeability [86,87]. The epithelial layer comprises tight junctions, adherens junctions, and desmosomes. Among TJs, claudins, occludin, and zonula occludens 1 (ZO-1) are critical for sealing the paracellular space [130]. Nrf2 activation enhances the expression of TJ proteins, including Claudin-4 and Occludin, through ARE-mediated transcription [76,131]. For example, activation of the ERK/Nrf2/HO-1 axis leads to increased levels of Occludin and ZO-1, whereas Nrf2-deficient mice exhibit downregulation of Claudin-4 and greater susceptibility to epithelial disruption [76,90].

Beyond transcriptional activation of junctional proteins, Nrf2 contributes to barrier maintenance via indirect mechanisms such as prevention of epithelial apoptosis and promotion of autophagy [76]. Recent studies have demonstrated that Nrf2-induced autophagy facilitates degradation of Claudin-2, a pore-forming TJ protein upregulated in IBD and associated with barrier leakiness [132]. Additionally, Nrf2 protects mitochondrial DNA, suppresses mitochondrial ROS, and reduces epithelial apoptosis in ischemia/reperfusion models, further underscoring its role in preserving epithelial viability and metabolic stability [133].

Nrf2 also regulates intestinal contractility by modulating signaling pathways in smooth muscle cells [134]. It influences acetylcholine receptor signaling, calcium flux, and the activity of second messengers [90,134]. Mice lacking Nrf2 show increased oxidation of tetrahydrobiopterin, reduced nitric oxide bioavailability, and impaired nitrergic relaxation, pointing to a role in neuromuscular coordination and gastric motility. Such changes may predispose to gastroparesis and delayed transit [90,134].

**Figure 5:**
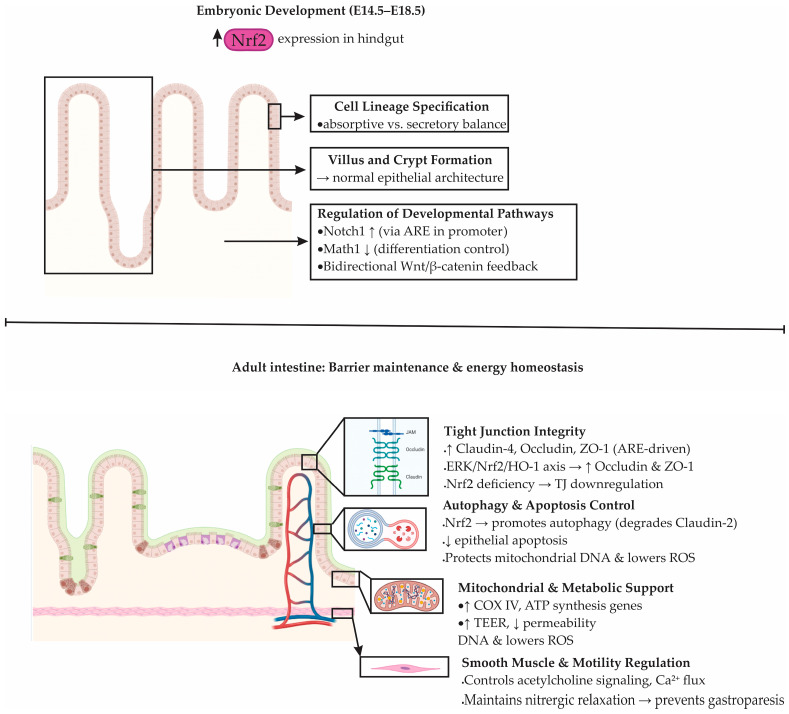
Physiological role of Nrf2 in intestinal development, epithelial homeostasis, and barrier maintenance. During embryogenesis (E14.5–E18.5), Nrf2 regulates villus–crypt formation, epithelial lineage balance, and developmental pathways including Notch1, Math1, and Wnt/β-catenin. In the adult intestine, Nrf2 maintains homeostasis by supporting tight-junction (TJ) integrity through the ERK/Nrf2/HO-1 pathway (ERK: extracellular signal-regulated kinase; HO-1: heme oxygenase-1), promoting autophagy and limiting apoptosis, protecting mitochondrial DNA, and reducing ROS (reactive oxygen species). Nrf2 also enhances ATP production and transepithelial electrical resistance (TEER) while regulating acetylcholine signaling and Ca^2+^ flux in smooth muscle to ensure proper motility.

Moreover, recent findings implicate Nrf2 in the regulation of energy metabolism within the gastrointestinal epithelium [135]. In mice lacking Nrf2, the expression of mitochondrial proteins such as cytochrome c oxidase subunit IV (COX IV) and genes responsible for adenosine triphosphate (ATP) production is reduced, impairing the energy-dependent maintenance of tight junctions [136]. This is accompanied by increased DNA oxidative damage, reduced transepithelial electrical resistance (TEER), and enhanced permeability in models of gastroesophageal reflux disease (GERD) [136]. ChIP assays confirmed direct binding of Nrf2 to the Claudin-4 promoter, linking redox control to epithelial bioenergetics and structural integrity [136]. The developmental and physiological roles of Nrf2 in intestinal morphogenesis, barrier maintenance, and metabolic regulation are summarized in Figure 5.

## 6. Crosstalk Between Nrf2 and Inflammatory Pathways in IBD: Mechanistic Insights and Cellular Context

Chronic intestinal inflammation, as seen in IBDs, is tightly linked to oxidative stress and redox imbalance [27]. The overproduction of ROS, a hallmark of intestinal inflammation, not only contributes to mucosal damage but also reinforces a cycle of persistent inflammation [27]. Reactive oxygen species activate redox-sensitive signaling pathways, including NF-κB, activator protein 1 (AP-1), and kinases such as MAPKs and phosphoinositide 3-kinase (PI3K). This activation stimulates the production of pro-inflammatory cytokines and further intensifies tissue injury [74,137]. This self-perpetuating cycle highlights the need for regulatory systems that can restrain oxidative stress and inflammatory signaling. Among these, the Nrf2-Keap1 pathway has emerged as a pivotal regulator of intestinal redox and immune homeostasis [76].

Upon oxidative insult, Nrf2 dissociates from its inhibitor Keap1 and translocates to the nucleus, where it orchestrates the expression of antioxidant enzymes such as HO-1, NQO1, and glutathione-related enzymes. These enzymes detoxify ROS and re-establish cellular homeostasis [76,138]. Notably, Nrf2 also indirectly suppresses inflammatory cascades by reducing ROS-mediated activation of NF-κB and by modulating upstream regulators such as IKKβ [139]. For example, Keap1 can interact with IKKβ, inhibiting IκBα degradation and preventing NF-κB nuclear translocation, thus attenuating the transcription of pro-inflammatory genes [139,140]. These regulatory loops position Nrf2 as both a sensor and modulator of intestinal inflammation.

In addition to its role in reducing inflammation, Nrf2 also regulates the polarization of immune cells. In macrophages, activation of Nrf2 decreases the pro-inflammatory M1 phenotype by lowering the expression of toll-like receptor 4 (TLR4), forkhead box protein O1 (FOXO1), and cytokines such as interleukin 1 beta (IL-1β) and interferon beta (IFN-β), while promoting the anti-inflammatory M2 phenotype. Through this dual action, Nrf2 helps limit tissue injury and supports the resolution of inflammation [141,142]. In contrast, Nrf2-deficient neutrophils exhibit exaggerated responses to LPS, with elevated levels of monocyte chemoattractant protein 1 (MCP-1), TNF-α, and ROS, further linking Nrf2 to immune regulation at the mucosal interface [142].

In addition to modulating cytokine production, Nrf2 regulates other inflammation-associated molecules, such as iNOS and COX-2, both of which are upregulated during intestinal inflammation. Studies show that Nrf2 activation suppresses the transcription of these enzymes in macrophages and dendritic cells [94,143,144]. Moreover, Nrf2 also affects extracellular matrix remodeling by regulating metalloproteinases (MMPs). Overexpression of NRF2 leads to reduced MMP-13 and MMP-9 levels, which has been linked not only to amelioration of inflammation in arthritis and ocular models but also to decreased cancer cell invasiveness via the Nrf2/HO-1 axis [145].

### 6.1. Nrf2 as a Key Guardian of the Intestinal Epithelial Barrier

The intestinal mucosal barrier is a complex defense system composed of mucus, epithelial cells, intercellular junctions, and commensal microbiota [130]. This multilayered structure separates luminal microbiota and toxins from host tissues, while still allowing controlled absorption of nutrients and water. Tight junctions, composed of proteins such as occludin, claudins, and members of the zonula occludens family, play a crucial role in controlling epithelial permeability and maintaining barrier integrity [130]. When barrier integrity is compromised, harmful substances and pathogens cross into the lamina propria, triggering immune activation and chronic inflammation. Such events are well documented in UC, where disruption of tight junctions amplifies mucosal injury and drives ulcer formation [146,147].

Mounting evidence shows that Nrf2 is indispensable for maintaining epithelial integrity. In models of DSS-induced colitis, expression of TJ proteins such as ZO-1 and claudin is markedly reduced, leading to increased permeability. Nrf2 activation restores their expression, thereby stabilizing the epithelial barrier [148,149]. Similarly, in LPS-induced epithelial damage, mitochondria-targeted antioxidants like MitoQ protect the barrier by stimulating Nrf2-dependent gene expression and reducing oxidative stress. These protective effects extend beyond the intestine [150]. In reflux esophagitis, Nrf2 binds directly to the claudin-4 promoter, enhancing its expression, whereas Nrf2 deficiency results in mitochondrial dysfunction, reduced claudin-4 levels, and impaired junctional integrity [136].

Nrf2 protects tight junctions not only by transcriptional regulation but also by suppressing epithelial apoptosis and promoting autophagy [94]. Mechanistic studies have shown that activation of the ERK/Nrf2/HO-1 axis triggers mitophagy, which alleviates oxidative stress and reinforces junctional proteins like ZO-1 and occluding [151]. Modulators of autophagy strongly influence this protective response, highlighting the dual role of Nrf2 in redox regulation and cellular quality control [122].

The functional role of Nrf2 in barrier defense has been validated across diverse disease models. In colonic inflammation induced by chronic kidney disease, treatment with the Nrf2 activator dh404 restored levels of occludin, claudin-1, and ZO-1, thereby repairing barrier integrity [152]. Likewise, in experimental colitis and systemic inflammatory models, genetic loss of Nrf2 consistently exacerbates permeability defects, whereas pharmacological or dietary activation of the pathway improves mucosal function [122].

### 6.2. Nrf2 as a Central Regulator of Intestinal Immune Homeostasis

The intestinal immune system plays a pivotal role in maintaining gut homeostasis, and its dysregulation is a hallmark of IBD [153,154]. Among the key molecular regulators in this context, Nrf2 has emerged as a critical modulator of both redox balance and immune cell function in the gastrointestinal tract [87].

Recent studies indicate that the activity of Nrf2 is closely linked to the differentiation and function of T helper (Th) cells [155,156,157]. When cluster of differentiation 4 positive (CD4^+^) T cells are activated, they develop into specialized subsets, including T helper 1 (Th1), T helper 2 (Th2), T helper 17 (Th17), and regulatory T cells (Tregs), each playing a distinct role in orchestrating the immune and inflammatory response [158]. Several studies have shown that Nrf2 activation skews this differentiation, favoring Th2 over Th1 responses [155,159,160]. For instance, the synthetic antioxidant tert-butylhydroquinone (tBHQ) suppresses Th1 cytokines like IFN-γ while promoting the expression of Th2-associated cytokines, such as IL-4 and IL-5 [159]. Moreover, Nrf2 activation appears to enhance IL-2 production, which is essential not only for Th2 polarization but also for the survival and stability of Treg cells, which help to maintain mucosal tolerance [160]. This suggests a dual immunoregulatory role for Nrf2, both in limiting excessive inflammation and in reinforcing epithelial tolerance mechanisms. Furthermore, through interaction with the aryl hydrocarbon receptor (AhR) pathway, Nrf2 can modulate IL-22 production in T cells, reinforcing its role in both adaptive immunity and mucosal defense [161].

Dimethyl fumarate (DMF), a known Nrf2 activator, has shown promise in experimental colitis models, where it reduced inflammation and improved histological outcomes [162,163,164]. These effects are believed to stem from simultaneous activation of antioxidant defenses and inhibition of the NF-κB pathway, a major driver of pro-inflammatory gene expression [162,163,164]. In an acute graft-versus-host disease model, DMF promoted the expansion of donor-derived Tregs and mitigated harmful allogeneic responses, further supporting the idea that Nrf2 shapes immune tolerance [165].

The role of Nrf2 is not limited to CD4^+^ T cells. CD8^+^ cytotoxic T lymphocytes (CTLs) also depend on Nrf2-mediated redox control for optimal function [166]. Loss of Nrf2 in macrophages impairs the antigen-driven activation of CD8^+^ T cells by limiting intracellular glutathione and cysteine levels, both essential for T cell metabolism and cytolytic activity [166]. Beyond T cells, Nrf2 also influences B cell function. Evidence indicates that B cells exposed to LPS increase immunoglobulin M (IgM) secretion when Nrf2 is pharmacologically activated [167]. Furthermore, Nrf2 signaling impacts dendritic cells, with its activation associated with suppressed antigen presentation capacity [168]. This dampening of dendritic cell activity indirectly favors T cell tolerance, while Nrf2 deficiency in these cells enhances Th1 polarization and T cell proliferation [168].

The immunoregulatory landscape also includes myeloid-derived suppressor cells (MDSCs), whose expansion and suppressive function are enhanced through Nrf2 activation [169]. In inflamed tissues, this contributes to reduced effector T cell activity and supports mucosal immune tolerance.

However, the immunoregulatory role of Nrf2 is context-dependent. Studies using genetically modified mice with constitutively active Nrf2 expression in epithelial or myeloid cells revealed exacerbated inflammation in acute colitis, but not in chronic models [170]. This paradox underscores the need for tight redox regulation, as both insufficient and excessive antioxidant responses can disturb mucosal immunity.

In clinical settings, findings remain somewhat inconsistent. Early research linked certain Nrf2 gene polymorphisms to UC susceptibility, particularly in females with a chronic disease phenotype [171]. Some studies reported reduced Nrf2 expression in patients with active UC and CD, while others found upregulated Nrf2 protein in inflamed mucosa [87,172]. These discrepancies may reflect differences in disease stage, sampling site, or compensatory responses to chronic oxidative stress. Interestingly, elevated Nrf2 in active disease correlated with increased expression of protective enzymes and inversely with pro-inflammatory IL-17A levels [173].

Current evidence on Nrf2 in CD is still limited. Some studies report that Nrf2 expression is reduced during active disease, while others demonstrate that pharmacological activation of Nrf2, for example with Bryostatin-1, alleviates colitis in interleukin 10 (IL-10)–deficient mice, an experimental model that mimics Crohn’s disease [174,175,176]. These observations suggest that Nrf2 may also hold therapeutic relevance in Crohn’s disease, although further studies are required.

### 6.3. Nrf2 as a Mediator of Gut Microbiota Balance and Intestinal Homeostasis

The gut microbiota constitutes a complex and dynamic ecosystem that plays a fundamental role in regulating host immunity, metabolism, and the integrity of the intestinal mucosa. In healthy individuals, this microbial community, although diverse in its composition, maintains a functional equilibrium that supports intestinal and systemic homeostasis [177,178]. However, microbial dysbiosis, characterized by disrupted composition and metabolite profiles, has been strongly implicated in the pathogenesis of IBD, colorectal cancer, and other intestinal disorders [179,180,181]. In IBD, dysbiosis is often marked by reduced microbial diversity, decreased abundance of beneficial Firmicutes (e.g., *Clostridiales*), and increased levels of potentially pathogenic Proteobacteria and Actinobacteria [182,183]. These shifts are associated with disease severity and mucosal barrier dysfunction.

Emerging evidence indicates that Nrf2 signaling exerts a profound influence on the gut microbial ecosystem, serving as a molecular interface between oxidative stress responses and microbial regulation [90,184]. Activation of Nrf2 can both shape microbial composition and mediate host tolerance to microbial metabolites [184]. For instance, oxidative stress in the gut can lead to epithelial barrier compromise, favoring dysbiosis; conversely, Nrf2 activation enhances antioxidant defense, preserving epithelial integrity and microbial balance [67,184,185].

### 6.4. Probiotics, Nrf2 Activation, and Gut Barrier Preservation

Several probiotic strains have demonstrated the ability to modulate the Nrf2 pathway, thereby exerting protective effects on the gut epithelium. For example, *Lactobacillus fermentum* Lf1 increased the expression of Nrf2 and downstream antioxidant enzymes such as SOD2 and thioredoxin reductase 1 (TrxR-1), while reducing lipid peroxidation in a DSS-induced colitis model [186]. Similarly, *Lactobacillus casei* Shirota protected epithelial cells against oxidative stress by activating both Nrf2 and NF-κB signaling pathways [187].

Other strains, such as *L. reuteri*, have been shown to enhance tight junction protein expression (e.g., occludin, claudin-1) and maintain mucosal barrier integrity via Nrf2/HO-1 axis activation [188]. In parallel, *Saccharomyces boulardii* alleviated colonic oxidative damage in murine colitis models through upregulation of SOD and catalase via Nrf2-mediated mechanisms [189]. These findings emphasize the dual role of probiotics in maintaining intestinal health. They not only modulate the redox balance through activation of antioxidant pathways but also reshape microbial communities, thereby helping to prevent or reduce colonic inflammation.

### 6.5. Dietary Compounds and Microbial Intermediates Acting Through Nrf2

Beyond live bacteria, bacterial metabolites and diet-derived phytochemicals also interface with the Nrf2 pathway [144]. For instance, Urolithin A (UroA), a microbial metabolite from ellagitannins in pomegranate and berries, activates the AhR-Nrf2 axis, leading to increased expression of antioxidant enzymes such as HO-1 and NQO1, and improved gut barrier function [184]. Similarly, Indole-3-lactic acid (ILA), produced by *Bifidobacterium longum* subsp. *infantis*, has been shown to reduce inflammation in epithelial cells through co-activation of AhR and Nrf2, enhancing the expression of target genes like GPX2, SOD2, and NQO1 [190].

Selenium nanoparticles (SeNPs) biosynthesized by *Lactobacillus casei* ATCC 393 further exemplify the therapeutic potential of microbiota–Nrf2 crosstalk [191]. These SeNPs improved epithelial antioxidant responses and tight junction integrity both in vitro and in DSS-induced injury models, underscoring a promising bioengineered approach to intestinal redox regulation [191].

Furthermore, *Lactobacillus helveticus*, *L. mali*, and other *Lactobacillus* strains have shown systemic effects beyond the gut, modulating hepatic oxidative stress and influencing the *Firmicutes*/*Bacteroidetes* ratio in high-fat diet models through Nrf2-related signaling [192,193]. The synergistic action of rice protein peptides and phytochemicals such as those from *Bruguiera gymnorrhiza* and *Tetrastigma hemsleyanum* have also been shown to activate the Keap1-Nrf2 pathway, enrich beneficial microbial taxa (e.g., *Akkermansia*, *Bifidobacterium*, *Anaerotruncus*), and enhance mucosal tight junction protein expression, suggesting diet–microbiota–host triad interactions mediated by redox-sensitive transcription factors [194].

To clarify these diverse functions, Table 2 summarizes the major Nrf2-dependent pathways implicated in IBD pathogenesis, emphasizing their cellular context and therapeutic relevance. 

## 7. Nrf2 in IBD-Associated Tumorigenesis: A Double-Edged Sword

Nrf2 is a central transcription factor controlling the expression of genes involved in antioxidant defense, detoxification, xenobiotic metabolism, and cellular stress responses [82]. Through these functions, Nrf2 preserves epithelial integrity and modulates inflammation, but its role in cancer biology is complex [195,196]. In the intestine, the nature and timing of Nrf2 activation largely determine whether it acts as a tumor suppressor or a tumor promoter [197]. Basal Nrf2 activity protects tissues from genotoxic insults, while sustained or dysregulated activation can favor tumor progression and chemoresistance [94,197,198].

Colorectal cancer (CRC) is one of the most common malignancies worldwide, and its incidence is significantly increased in patients with long-standing ulcerative colitis [199]. The chronic inflammatory microenvironment characteristic of IBD, rich in ROS, cytokines, and infiltrating immune cells, fosters mutagenesis and epigenetic changes that drive malignant transformation [200,201]. Nrf2 regulates many cytoprotective genes that mitigate oxidative and proteotoxic stress, and in early stages this activity may counteract DNA damage and reduce tumor initiation [197,202]. However, persistent Nrf2 activation may inadvertently support the survival of dysplastic or transformed cells by suppressing apoptosis, maintaining proteasome activity, and enhancing DNA repair [203,204].

Experimental models illustrate this duality. In DSS-induced colitis-associated cancer, Nrf2 deficiency produced opposing outcomes depending on disease stage. Some studies showed that Nrf2 knockout mice developed more numerous and larger tumors with increased inflammatory mediators such as COX-2, lipoxygenases, and prostaglandins, highlighting its protective function during early carcinogenesis [89,205]. In contrast, other models demonstrated that ablation of Nrf2 reduced aggressive tumor formation and upregulated tumor suppressors such as 15-hydroxyprostaglandin dehydrogenase (15-PGDH), suggesting that in later stages Nrf2 may facilitate tumor growth [89,205]. These conflicting results underscore that the impact of Nrf2 on tumorigenesis is context-dependent.

Clinical and mechanistic data further support this dual role. Polymorphisms in the Nrf2 promoter region have been linked to higher CRC risk, while high nuclear expression of Nrf2 correlates with increased antioxidant enzyme levels and lower inflammatory markers in colonic mucosa [206,207]. Conversely, cytoplasmic retention of Nrf2 (cNrf2) in tumor cells is associated with poor prognosis, increased metastasis, and resistance to chemotherapy [208]. Mechanistically, cNrf2 interacts with PSMD4 to promote nuclear export and activate downstream pathways such as NF-κB, AKT, and β-catenin, conferring resistance to 5-fluorouracil (5-FU) and oxaliplatin [208]. Epigenetic changes, such as DNA demethylation, may also upregulate Nrf2 and HO-1 expression in drug-resistant CRC cells, further contributing to chemoresistance [209,210].

Nrf2 also influences key processes in metastasis. Its overexpression in highly invasive CRC correlates with advanced Duke’s stage and increased vascular endothelial growth factor (VEGF) signaling [211]. Nrf2 enhances angiogenesis under hypoxic conditions by stabilizing hypoxia-inducible factor 1 alpha (HIF-1α) and inducing VEGF expression, whereas inhibition of Nrf2 reduces tumor vascularization and growth in xenograft models [212]. These findings suggest that while Nrf2 activation may be beneficial in preventing oxidative damage in premalignant tissues, its persistent activation in established tumors can facilitate angiogenesis, invasion, and therapy resistance.

## 8. Nrf2-Linked Regulation of Intestinal Fibrosis

Fibrosis represents one of the most challenging complications in inflammatory bowel diseases, particularly CD, where it leads to stricture formation and bowel obstruction [213]. Unlike ulcerative colitis, where fibrosis is mostly confined to the mucosa and submucosa, in CD the remodeling process involves the entire bowel wall [213]. This remodeling is driven by chronic inflammation, persistent epithelial injury, and an imbalance between extracellular matrix (ECM) deposition and degradation [214]. Excessive accumulation of ECM proteins such as collagen thickens and stiffens the intestinal wall, contributing to stricture development and functional impairment [215].

At the molecular level, TGF-β is recognized as a central mediator of fibrogenesis. It stimulates fibroblast-to-myofibroblast transition, activates collagen-producing cells, and orchestrates ECM accumulation [215,216]. TGF-β signaling operates mainly through the Smad pathway but also intersects with MAPK cascades [217]. Importantly, the activity of TGF-β is closely tied to redox homeostasis. Elevated levels of ROS can amplify TGF-β/Smad signaling, creating a feed-forward loop that enhances fibrotic responses [218,219].

Nrf2 plays a critical role in counteracting these processes. By inducing antioxidant enzymes such as HO-1, NQO1, and SOD, Nrf2 limits oxidative stress and suppresses pro-fibrotic signaling [76]. Experimental studies in TNBS-induced colitis have shown that pharmacological activation of Nrf2 with tBHQ reduces fibrosis by inhibiting the TGF-β/Smad pathway [220,221]. In contrast, Nrf2 deficiency enhances fibroblast sensitivity to TGF-β, promoting their differentiation into myofibroblasts and driving ECM overproduction [220]. This demonstrates a protective, anti-fibrotic role of Nrf2 through regulation of the ROS–TGF-β–Smad axis.

In addition to TGF-β signaling, the balance between MMPs and their inhibitors (TIMPs) is crucial for ECM turnover. Dysregulation of this system accelerates fibrosis [215]. Among MMPs, MMP-7 and MMP-3 appear particularly relevant in IBD [222]. The Nrf2/HO-1 pathway has been shown to suppress MMP-7 activity in intestinal epithelial cells, suggesting a direct mechanism by which Nrf2 curtails fibrotic remodeling [87,223]. Elevated MMP-3 levels have been detected in both CD and UC patients, with concentrations correlating with disease severity and response to anti-TNF therapy [224,225]. Notably, Nrf2-deficient mice exhibit exaggerated MMP-3 expression, linking oxidative stress with impaired ECM regulation [226].

Although fibrosis is less common in UC, evidence suggests that submucosal thickening and muscularis mucosae remodeling can occur, particularly in patients with long-standing disease. These findings underscore the need to better understand the drivers of fibrosis in both forms of IBD and highlight the potential of targeting redox-sensitive pathways. Nrf2, by integrating ROS detoxification with regulation of fibrotic signaling, emerges as a promising therapeutic node. Agents capable of fine-tuning Nrf2 activation may help restore ECM balance, limit fibrotic complications, and ultimately improve long-term outcomes in IBD. Table 3 summarizes the principal Nrf2-dependent pathways implicated in IBD-associated tumorigenesis and intestinal fibrosis, highlighting their molecular targets and clinical relevance

## 9. Pharmacological and Nutritional Modulation of the Nrf2/Keap1 Pathway in the Treatment of Inflammatory Bowel Disease

Over recent years, the Nrf2/Keap1 signaling axis has emerged as a promising therapeutic target in IBD, including both Crohn’s disease and ulcerative colitis [94,227]. This transcriptional system regulates cellular responses to oxidative stress by inducing a battery of cytoprotective genes involved in antioxidant defense, inflammation control, and epithelial integrity maintenance [142,198]. Regulation of this pathway, whether through pharmacological agents or naturally occurring bioactive compounds, has demonstrated beneficial effects in experimental models of colitis [227,228].

Among clinically approved drugs, 5-aminosalicylic acid (5-ASA) remains a cornerstone in IBD therapy [229]. Beyond its anti-inflammatory activity, 5-ASA enhances Nrf2 nuclear translocation in inflamed colonic mucosa, leading to increased expression of HO-1 and suppression of TNF-α [230,231]. This redox-sensitive activation appears to occur selectively under inflammatory conditions, suggesting that 5-ASA may function as a prodrug whose efficacy depends on local oxidative stress. Dimethyl fumarate (DMF), currently used for multiple sclerosis, has similarly demonstrated the ability to activate Nrf2 and suppress colonic inflammation in animal models [162].

Other repurposed agents such as telmisartan, olmesartan, and dapagliflozin also exert protective effects in experimental colitis via Nrf2 activation [94]. These drugs reduce oxidative injury and inflammatory cytokine production while promoting the expression of detoxifying enzymes [94]. Notably, metformin, traditionally used in type 2 diabetes, enhances Nrf2 activity through AMPK-dependent mechanisms, reinforcing the interconnectedness between metabolic and redox pathways in IBD pathology [232]. Novel compounds like CPUY192018 directly target Keap1 to stabilize Nrf2 and are currently under investigation as potential disease-modifying agents [233].

In parallel, numerous naturally derived compounds have gained attention as Nrf2 inducers with therapeutic potential in IBD. Flavonoids such as luteolin, galangin, genistein, and puerarin have shown to upregulate HO-1 and NQO1 in DSS-induced colitis, resulting in reduced oxidative stress and improved mucosal architecture [144]. Terpenoids, particularly ginsenosides and compounds such as dehydrocostus lactone, modulate the Nrf2/HO-1 axis while suppressing NF-κB and apoptotic mediators, thereby preserving epithelial barrier function [234]. Alkaloids like berberine and rutaecarpine demonstrate similar benefits, acting through Nrf2-mediated regulation of P-glycoprotein and antioxidant enzymes [235,236].

Polysaccharides from herbal sources such as Astragalus membranaceus and Aloe vera have also shown strong efficacy in restoring epithelial integrity and lowering oxidative markers in preclinical models [94]. These complex carbohydrates activate Nrf2 signaling and suppress ferroptosis and pyroptosis, two forms of regulated cell death that are increasingly recognized as key contributors to the development of inflammatory bowel disease IBD [94].

Several medicinal plant extracts, including Perilla frutescens, Forsythia suspensa, Artemisia argyi, and Vaccinium species, have demonstrated the capacity to activate Nrf2, often in conjunction with suppression of NF-κB and MAPK pathways [144]. These extracts not only modulate inflammation but also improve tight junction integrity and microbiota composition.

Traditional Chinese medicine (TCM) formulations, such as Huang-Lian-Jie-Du decoction and Banxia Xiexin decoction, offer multi-target therapeutic strategies [149,237]. Their synergistic components activate Nrf2 while simultaneously suppressing pro-inflammatory transcription factors, offering an integrative approach to IBD therapy.

## 10. Comparative Perspective: Nrf2 Versus Galectin-3 as Emerging Biomarkers in IBD

Nrf2 and galectin-3 (Gal-3) represent two mechanistically distinct biomarker families that capture different pathogenic dimensions of inflammatory bowel disease [87,238]. Nrf2 functions as a transcriptional regulator of antioxidant and cytoprotective genes, offering an upstream readout of redox imbalance and epithelial stress [87]. By contrast, Gal-3 is a secreted lectin measurable in serum and tissue that reflects innate immune activation, macrophage activity, and early fibrotic remodeling [238].

Clinically, Nrf2-related signatures correlate with oxidative stress, impaired barrier function, and shifts in mucosal antioxidant capacity—parameters not captured by routine inflammatory markers such as CRP or fecal calprotectin [87]. Gal-3, on the other hand, rises with endoscopic severity, predicts fibrotic complications, and associates with response to anti-TNF therapy, highlighting its translational usefulness as a soluble biomarker [238]. 

Mechanistically, Nrf2 suppresses ROS-driven NF-κB activation, stabilizes tight junctions, and modulates immune polarization, positioning it as a regulator of upstream oxidative and inflammatory loops [87]. Gal-3 primarily amplifies leukocyte recruitment, macrophage activation, and fibroblast proliferation, linking it more directly to chronic inflammation and tissue remodeling [238].

Therapeutically, Nrf2 is already a validated drug target, with activators such as dimethyl fumarate showing efficacy in experimental colitis. [87] Gal-3 inhibitors are emerging but remain earlier in development and less explored in IBD [238].

Together, Nrf2 and Gal-3 highlight complementary aspects of IBD pathogenesis—redox imbalance versus lectin-mediated inflammation—and combined assessment may ultimately offer improved disease stratification and prediction of complications. 

## 11. Future Directions in Nrf2 Modulation and Its Therapeutic Potential in IBD

Despite the strong preclinical evidence supporting Nrf2 as a therapeutic target in IBD, multiple challenges limit the clinical translation of Nrf2-modulating strategies. One of the key barriers is the context-dependent nature of Nrf2 activation, which can be protective in early inflammation but potentially detrimental in chronic disease stages or in the presence of dysplastic lesions. Sustained or excessive Nrf2 activation may promote epithelial survival, impair apoptosis of damaged cells, and contribute to chemoresistance or tumor progression. This duality underscores the need to define disease stage–specific therapeutic windows and to develop pharmacologic agents capable of precise, titratable Nrf2 activation.

Another major challenge is the lack of reliable biomarkers for monitoring Nrf2 pathway activity in vivo. Current studies rely on transcript levels of downstream enzymes such as HO-1 or NQO1, but these readouts lack temporal sensitivity and may be influenced by parallel inflammatory pathways. To implement Nrf2-directed therapies safely, future studies should identify non-invasive biomarkers (e.g., metabolomic signatures, redox-derived lipids, circulating antioxidant capacity markers) that reflect real-time pathway activation and help stratify patients who may benefit from targeted interventions.

A further obstacle concerns the heterogeneity of IBD phenotypes, which may result in differential Nrf2 responses across patients. Variants in the NFE2L2 promoter, differences in microbiota composition, and variability in epithelial redox status may all influence responsiveness to Nrf2-activating drugs. Precision-medicine approaches integrating genetic stratification, microbiome profiling, and redox phenotyping will be essential to determine which patient subgroups derive the greatest benefit.

Additionally, while numerous phytochemicals and probiotics show Nrf2-activating properties, their bioavailability, metabolic stability, and tissue-specific targeting remain inconsistently characterized. The development of optimized formulations—such as nanoencapsulation, targeted delivery systems, or engineered probiotic strains—could enhance mucosal uptake and reduce systemic off-target effects. Similarly, selective Keap1 inhibitors and next-generation small molecules should be evaluated in long-term models to assess potential safety concerns, particularly regarding tumorigenesis.

Finally, further work is needed to clarify how Nrf2 interacts with other redox-sensitive pathways, including NF-κB, STAT3, ferroptosis regulators, and mitochondrial quality-control mechanisms. Understanding these interactions may allow design of rational combination therapies, where Nrf2 activation is paired with immunomodulators, microbiota-directed therapies, or anti-fibrotic agents. Longitudinal human studies integrating multi-omics, functional imaging, and organoid systems will be instrumental in defining how the Nrf2 network evolves across disease stages and treatment responses.

Together, these lines of inquiry point to an urgent need for biomarker-guided, context-specific, and precision-engineered approaches to safely leverage Nrf2 signaling as a therapeutic axis in IBD.

## 12. Conclusions

The Nrf2/Keap1 signaling pathway plays a multifaceted and dynamic role in the pathophysiology of inflammatory bowel disease. As a master regulator of antioxidant defense, Nrf2 not only neutralizes reactive oxygen species and limits oxidative injury but also orchestrates immune responses, reinforces the epithelial barrier, and shapes the intestinal microbiome. Its protective functions are context-dependent—conferring cytoprotection in early disease stages, yet potentially promoting tumorigenesis and fibrosis under conditions of sustained or dysregulated activation. Preclinical and clinical data underscore the translational potential of targeted Nrf2 modulation, whether through pharmacological activators, repurposed drugs, or dietary and probiotic interventions. However, fine-tuning the intensity and timing of Nrf2 activation remains essential to avoid adverse effects and achieve therapeutic precision. Future research should focus on biomarker-guided patient stratification and the development of Nrf2-based combinatorial therapies that complement existing immunomodulatory approaches in IBD.

## Figures and Tables

**Table 1:** Nrf2–Keap1 pathway: activation mechanisms, molecular targets, and therapeutic significance in inflammatory bowel disease.

Mechanism/Component	Key Molecular Events and Targets	Clinical Significance/Therapeutic Implication	References
Basal regulation (Keap1–Cul3 complex)	Keap1 binds Nrf2 via DLG/ETGE motifs, promoting ubiquitination and proteasomal degradation under homeostatic conditions	Maintains low Nrf2 levels; ensures rapid activation only during stress; dysregulation increases susceptibility to oxidative injury	[72,73,98,99,100]
Keap1-dependent activation	Oxidation of cysteine residues (Cys151, Cys273, Cys288) in Keap1 disrupts its interaction with Nrf2, halting ubiquitination and enabling nuclear translocation	Central antioxidant defense mechanism; potential for targeting Keap1 cysteines by electrophilic drugs or natural compounds (e.g., sulforaphane)	[74,99,100,101,102,103,104,105,106]
Keap1-independent activation (kinase-mediated)	Phosphorylation of Nrf2 by PKC (Ser40), AMPK (Ser550), and MAPKs (ERK, JNK, p38) enhances Nrf2 stability and transcriptional activity	Provides flexibility to integrate redox and metabolic signals; pharmacologic AMPK activators and PKC modulators enhance mucosal protection	[107,108,109,110,111,112,113,114,115,117]
Transcriptional activation (ARE binding)	Nrf2 heterodimerizes with small Maf proteins and binds to AREs, inducing genes such as HO-1, NQO1, SOD, GPx, and GCL	Drives antioxidant, detoxifying, and cytoprotective responses; supports tissue regeneration and redox homeostasis	[74,75,76,88,100]
Anti-inflammatory and barrier-protective functions	Nrf2 suppresses NF-κB and iNOS signaling, limits cytokine production (TNF-α, IL-1β, IL-6), and preserves epithelial integrity	Reduces mucosal inflammation, promotes healing, and may synergize with immunosuppressants in IBD management	[77,78,79,80,81,82,86,87]

**Table 2:** Key Nrf2-Dependent Pathways in Intestinal Inflammation, Barrier Regulation, and Microbial Homeostasis.

Key Mechanism/Pathway	Cell/Tissue Context	Clinical Significance/Therapeutic Implication	References
Nrf2–Keap1 antioxidant axis	Epithelium, macrophages	Restores redox balance, limits ROS-driven mucosal injury; pharmacological activators (e.g., DMF) show benefit in colitis models	[76,138,162,163,164]
Inhibition of NF-κB and redox-sensitive inflammatory signaling	Myeloid and epithelial cells	Reduces transcription of pro-inflammatory cytokines (TNF-α, IL-1β, IL-6); attenuates chronic inflammation	[139,140,141,142,143]
Maintenance of tight junctions (ZO-1, occludin, claudins)	Intestinal epithelial barrier	Nrf2 activation restores TJ protein expression and improves permeability in DSS/LPS models	[146,147,148,149,150,151,152]
Regulation of fibroblast activation (TGF-β/Smad axis)	Intestinal fibroblasts	Nrf2 suppresses ROS-driven TGF-β signaling, limiting myofibroblast differentiation and intestinal fibrosis	[113,114,115,117,118,120]
Modulation of metalloproteinases (MMP-3, MMP-7)	Epithelium, fibroblasts	Nrf2/HO-1 axis inhibits MMP-driven ECM remodeling; elevated MMP-3 predicts poor biologic response	[91,94,95,96,97]
Immune modulation (macrophage M1 → M2 shift; Th1/Th2/Treg balance)	Macrophages, CD4^+^ T cells	Supports anti-inflammatory phenotypes, enhances mucosal tolerance, reduces tissue injury	[141,155,156,157,158,159,160,161]
Interaction with gut microbiota	Epithelial–microbial interface	Nrf2 activation protects epithelial integrity, prevents dysbiosis, and maintains microbial–host homeostasis	[67,184,185]
Probiotic- and metabolite-induced Nrf2 activation	Epithelium	Lactobacillus and microbial metabolites (UroA, ILA, SeNPs) enhance antioxidant defenses and strengthen the barrier	[186,187,188,189,190,191,192,193,194]

**Table 3:** Key Nrf2-Dependent Mechanisms in IBD-Associated Tumorigenesis and Fibrosis.

Mechanism/Pathway	Cell/Tissue Context	Clinical Significance/Therapeutic Implication	References
Basal Nrf2 antioxidant defense	Intestinal epithelium	Protects DNA from ROS injury; reduces early tumor initiation	[195,196,197,198,199,200,201,202]
Nrf2 suppression of inflammatory enzymes (COX-2, LOX, iNOS)	Inflamed mucosa; tumor-adjacent epithelium	Limits early carcinogenesis; Nrf2 deficiency increases tumor burden	[89,205]
Cytoplasmic Nrf2 (cNrf2) retention	Dysplastic & invasive CRC cells	Correlates with metastasis, poor prognosis, chemoresistance	[208,209,210]
Nrf2–HIF-1α–VEGF axis	Hypoxic tumor microenvironment	Promotes angiogenesis; Nrf2 inhibition reduces tumor vascularization	[211,212]
Nrf2 regulation of TGF-β/Smad signaling	Fibroblasts; myofibroblasts	Activation of Nrf2 suppresses fibrogenic signaling; reduces collagen deposition	[220,221]
ROS–TGF-β feed-forward loop	Inflamed and remodeling bowel wall	Nrf2 deficiency amplifies ROS-driven fibroblast activation and ECM accumulation	[218,219]
Nrf2/HO-1 inhibition of MMP-7	Intestinal epithelial cells	Reduces pathological ECM remodeling; potential target for anti-fibrotic therapy	[87,223]
Nrf2 control of MMP-3 expression	Intestinal mucosa; serum biomarkers	Elevated in UC/CD; predicts anti-TNF response; exaggerated in Nrf2-deficiency	[224,225,226]

## Data Availability

No new data were created or analyzed in this study. Data sharing is not applicable to this article.

## Abbreviations

The following abbreviations are used in this manuscript:

5-ASA	5-aminosalicylic acid
5-FU	fluorouracil
α-SMA	alpha-smooth muscle actin
AhR	aryl hydrocarbon receptor
AMPK	adenosine monophosphate–activated protein kinase
AP-1	activator protein 1
AREs	antioxidant response elements
ATP	adenosine triphosphate
bZIP	of basic leucine zipper
CBP	CREB-binding protein
CD	Crohn’s disease
CD4^+^	cluster of differentiation 4 positive
CNC	the Cap’n’Collar
COX IV	cytochrome c oxidase subunit IV
COX-2	cyclooxygenase-2
CRC	Colorectal cancer
CTLs	cytotoxic T lymphocytes
cNrf2	cytoplasmic retention of Nrf2
Cul3	cullin-3
DMF	Dimethyl fumarate
ECM	extracellular matrix
ERK	extracellular signal–regulated kinase
FOXO1	forkhead box protein O1
GCL	glutamate–cysteine ligase
GSH	glutathione
GST	glutathione S-transferase
HIF-1α	hypoxia-inducible factor 1 alpha
HO-1	heme oxygenase-1
HOCl	hypochlorous acid
IBD	Inflammatory bowel disease
IFN-β	interferon beta
IgM	immunoglobulin M
IKKβ	I kappa B kinase beta
ILA	Indole-3-lactic acid
IL-1β	interleukin 1 beta
IL-8	interleukin 8
iNOS	inducible nitric oxide synthase
JNK	c-Jun N-terminal kinase
Keap1	Kelch-like ECH-associated protein 1
Maf	musculoaponeurotic fibrosarcoma
MAPK	mitogen-activated protein kinase
MAPKs	mitogen-activated protein kinases
Mkp-1	mitogen-activated protein kinase phosphatase 1
MMPs	metalloproteinases
NF-κB	nuclear factor kappa B
NLRP3	NLR family pyrin domain containing 3
NQO1	NAD(P)H:quinone oxidoreductase 1
Nrf2	Nuclear factor erythroid 2–related factor 2
NSAIDs	nonsteroidal anti-inflammatory drugs
O_3_	ozone
PI3K	phosphoinositide 3-kinase
Pink1	phosphatase and tensin homolog–induced putative kinase 1
PKC	protein kinase C
Rbx1	RING-box protein 1
ROS	reactive oxygen species
SeNPs	Selenium nanoparticles
SOD	superoxide dismutase
STAT3	signal transducer and activator of transcription 3
TGF-β1	transforming growth factor beta 1
Th	T helper
TIMP-1	tissue inhibitor of metalloproteinases 1
TJs	tight junctions
TLR4	toll-like receptor 4
TNBS	trinitrobenzene sulfonic acid
TNF-α	tumor necrosis factor alpha
TrxR-1	thioredoxin reductase 1
Tregs	regulatory T cells
tBHQ	tert-butylhydroquinone
UC	ulcerative colitis
UGT1A1	uridine diphosphate glucuronosyltransferase 1A1
UroA	Urolithin A
VEGF	vascular endothelial growth factor
ZnO	Zinc oxide
ZO-1	zonula occludens 1
